# Knowledge, awareness, attitudes and practice regarding oral potentially malignant disorders and oral cancer among health professional students in Riyadh, Saudi Arabia: a multicenter cross-sectional study

**DOI:** 10.3389/froh.2026.1942387

**Published:** 2026-10-21

**Authors:** Sanjeev B. Khanagar, Adeem Alofi, Leen Abdulrahman Alzaaqi, Jawaher Mohammed Alsubaie, Dalal Alayed, Setah Mansour Abothnin, Aljwhrah Abdulaziz Almuammar, Areej Alfaifi, Kiran Iyer

**Affiliations:** 1 Preventive Dental Science Department, College of Dentistry, King Saud bin Abdulaziz University for Health Sciences, Riyadh, Saudi Arabia; 2 King Abdullah International Medical Research Centre, Riyadh, Saudi Arabia; 3 Ministry of the National Guard Health Affairs, Riyadh, Saudi Arabia; 4 College of Dentistry, King Saud bin Abdulaziz University for Health Sciences, Riyadh, Saudi Arabia; 5 Department of Restorative and Prosthetic Dental Sciences, College of Dentistry, King Saud bin Abdulaziz University for Health Sciences, Riyadh, Saudi Arabia

**Keywords:** attitudes, awareness, dental, early detection, health professional, healthcare, knowledge, oral cancer

## Abstract

**Background:**

Oral potentially malignant disorders (OPMDs) are known precursors of oral cancer (OC), and early detection of OPMDs is crucial for reducing disease-related morbidity and mortality. As future healthcare providers, health professional students must possess adequate knowledge and clinical preparedness for the prevention and early detection of OPMDs and OC. This study aimed to assess the knowledge, awareness, attitudes, and practices regarding OPMDs and OC among health professional students in Riyadh, Saudi Arabia.

**Methods:**

A multicenter cross-sectional study was conducted among undergraduate students in medicine, dentistry, nursing, and applied medical sciences in Riyadh, Saudi Arabia. Data were collected using a culturally adapted and content-validated questionnaire derived from a previously validated instrument which consisted of 32 items. Descriptive statistics, chi-square tests, cluster analysis, and binary logistic regression were employed to examine factors associated with knowledge, awareness, attitudes, and practices. A *p* < 0.05 was considered statistically significant.

**Results:**

A total of 456 students were included in the study. Two distinct knowledge clusters were identified, representing relatively higher and lower levels of knowledge regarding OPMDs and OC. Dental students showed higher proportions of knowledge, awareness, positive attitudes, and good clinical practices compared with other professional disciplines; however, these findings should be interpreted cautiously because the dental subgroup was small (*n* = 23). Logistic regression showed higher odds of belonging to the higher knowledge cluster among dental students (OR = 12.67, 95% CI: 4.17–38.40, *p* < 0.001), although the wide confidence interval indicates imprecision. Higher knowledge was significantly associated with greater awareness (OR = 2.07, 95% CI: 1.35–3.19, *p* = 0.001). High awareness and positive attitudes were also significantly associated with good clinical practices. Knowledge varied descriptively across the participating universities; however, the association did not reach conventional statistical significance in the present sample (*p* = 0.057). Given the substantial imbalance in university sample sizes, this finding should not be interpreted as evidence of equivalent knowledge levels or the absence of institution-specific educational differences.

**Conclusions:**

The findings demonstrated variation in knowledge, awareness, attitudes, and practices across different professional groups. Therefore, targeted educational interventions are essential to address these identified gaps.

## 1 Introduction

Oral cancer (OC) is a major health problem all over the world and one of the most common malignancies in the head and neck region (1, 2). Oral cancer includes cancers of the lip and oral cavity, including the tongue, gums, floor of the mouth, palate, and other parts of the mouth. According to the latest GLOBOCAN 2022 estimates, approximately 389,846 new cases and 188,438 deaths worldwide in 2022, ranking as the 16th most common cancer by incidence and the 15th leading cause of cancer-related mortality globally (3). Despite advances in prevention and treatment, oral cancer continues to impose a substantial global health burden, particularly in low- and middle-income countries, where its incidence and mortality rates remain disproportionately high. Most oral squamous cell carcinomas develop from oral potentially malignant disorders (OPMDs), formerly known as oral precancerous lesions (4). Precancerous lesions with different risks of malignant transformation include leukoplakia, erythroplakia, oral lichen planus, oral submucous fibrosis and tobacco pouch keratosis. OPMDs may affect up to half a billion people worldwide, and early detection and reduction of lifestyle risk factors are critical strategies to alleviate this serious burden (5). Early diagnosis and treatment of these lesions greatly improve outcome, reduces disease progression and increases patient survival. Therefore, knowledge and recognition of OPMDs are essential parts of OC prevention strategies.

The main goal of early detection of cancer is to reduce morbidity and mortality of cancer. The detection of precancerous lesions is considered as an important tool to achieve this goal (6). For instance, OPMDs are associated with increased risk of OC, and the detection of these early lesions has been considered an essential stage in the early diagnosis of cancer for a long time (7).

There are many factors that lead to the development of OC. Tobacco smoking, use of smokeless tobacco and alcohol drinking continue to be the most recognized risk factors worldwide. Other factors include infection with the human papilloma virus (HPV), chronic irritation to the mucosa, poor dietary habits, genetic predisposition and long exposure to ultraviolet radiation, especially in cancers of the lip (8, 9). Smoking and the use of smokeless tobacco products such as Shammah are still major contributors to the burden of OC in Saudi Arabia, particularly in certain regions of the country (10).

Healthcare professionals play an important role in the prevention, early detection and referral of patients with OPMDs and OC (2, 11). Routine clinical examination with risk assessment, patient counseling and appropriate referral at the right time can reduce the delay in diagnosis and improve the prognosis significantly. Hence, it is important for healthcare students to be well informed and knowledgeable during their undergraduate education to be efficient in identifying suspicious oral lesions and educating patients about modifiable risk factors in the future. However, previous studies have shown a wide variation in the knowledge and awareness of OC among healthcare students. Medical, nursing, pharmacy and allied health students generally have poor knowledge of early signs, symptoms and premalignant conditions, whereas dental students are generally more knowledgeable about risk factors and clinical features of OC (11–13). A recent systematic review showed that although awareness of tobacco-related risks was relatively high, the awareness of other risk factors and OPMDs is still inadequate among health professional students globally (14).

The existing literature in Saudi Arabia has mainly targeted dental students and the general population showing a moderate level of awareness of OC but with some gap in knowledge about early clinical features and preventive measures (11, 13). Insufficient knowledge and misconceptions about OC and its risk factors have also been reported in studies involving dental patients and general populations in Riyadh and Jazan. However, there is a lack of evidence in relation to multidisciplinary healthcare students (including dentistry, medicine, nursing, and applied medical sciences) especially in Riyadh where future healthcare professionals are trained in different academic settings.

Early detection is vital and health care providers have an important role in the prevention of OC. Therefore, a comprehensive assessment of knowledge, awareness, attitude and practices of health professional students towards OPMDs and OC is necessary. Identifying current educational gaps will provide valuable information to enhance curricula and develop targeted educational interventions to improve early detection, prevention and patient outcomes. The present study was designed to assess the knowledge, awareness, attitudes and practices of health professional students regarding OPMDs and OC in Riyadh, Saudi Arabia.

## 2 Materials and methods

### 2.1 Research design and setting

An analytical cross-sectional study was conducted in line with the Declaration of Helsinki. Prior to the commencement of the research, ethical approval was obtained from the Institutional Review Board of the King Abdullah International Medical Research Center, Riyadh, Saudi Arabia (KAIMRC) (IRB No. NRR26/104/1, approved on 24 February 2026).

This multicenter analytical cross-sectional study was conducted among undergraduate students enrolled in health professional colleges across universities in Riyadh, Saudi Arabia. The data collection for this study took place between February 2026 and May 2026. Health professional education in Saudi Arabia is offered by numerous governmental and private institutions in Riyadh, including programs in medicine, dentistry, nursing, and applied medical sciences. The study was carried out in accordance with the Strengthening the Reporting of Observational Studies in Epidemiology (STROBE) guidelines for cross-sectional studies (15). The details are presented in Supplementary Table 1.

### 2.2 Sample size estimation

The sample size was estimated based on results reported in the literature (12). It was calculated to achieve 90% power and a 95% confidence interval for a prevalence rate of 50%. Sample size estimation was conducted using G*Power software (version 3.1.9.4). The required sample size for this study is 394 participants, including both males and females.

Test: *χ*^2^ tests – Goodness-of-fit tests: Contingency tables
Analysis: *a priori* – Compute required sample size
Effect size (w): 0.20
*α* error probability: 0.05
Power (1−*β*): 0.90
*β* error probability: 0.10
Degrees of freedom: 5
Noncentrality parameter *λ*: 16.48
Critical *χ*^2^: 11.0705
Required total sample size: 412
Actual achieved power: 0.9002

### 2.3 Sampling technique

Riyadh was geographically divided into five regions to facilitate recruitment from health professional institutions. Geographically, Riyadh was divided into five regions: North, South, East, West and Central (University A, B, C, D, E). We identified universities in each region offering programs for health professionals. Eligible undergraduate students from participating universities were recruited through voluntary online convenience sampling. The survey link was distributed electronically via university mailing lists, student organizations, and academic coordinators, and was accessible to eligible students who voluntarily chose to participate. Since no individual-level sampling frame was available, students were not randomly selected. Therefore, the final sample represents only those students who voluntarily responded to the online questionnaire, rather than a probability-based sample of all eligible health professional students in Riyadh.

### 2.4 Eligibility criteria

Inclusion Criteria: Undergraduate health professional students who are enrolled in colleges located in Riyadh, Saudi Arabia. Students of Medicine, Dentistry, Nursing, and Medical Applied Sciences Students who are willing to participate and willing to sign the informed consent form.

Exclusion Criteria: Graduated health professionals. Students not currently enrolled in a health care program

### 2.5 Data collection tool

The questionnaire used in this study was modified from a previously validated questionnaire developed by Nawaz et al. (16). The original questionnaire exhibited good psychometric properties, with a scale-level content validity index (S-CVI/Ave) of 0.94, Cohen's kappa coefficient of 0.93, and a Cronbach's alpha coefficient of 0.82 (16).

For contextual and cultural adaptation to healthcare professional students in Saudi Arabia, the questionnaire was content validated by a panel of five experts (specialists in oral medicine, oral pathology, and dental public health) to ensure cultural relevance, and contextual appropriateness to the Saudi Arabia healthcare setting. The experts rated each item independently on a four-point rating scale (1 = not relevant, 2 = somewhat relevant, 3 = quite relevant, and 4 = highly relevant) for its relevance, clarity and cultural appropriateness.

The experts assessed each item for relevance, clarity, and cultural appropriateness. Based on expert advice and consensus, 22 items out of the original 54 items were removed due to their cultural inappropriateness, contextual irrelevance or duplication within the context of Saudi Arabia. Items addressing alcohol use, betel quid/areca nut/gutka chewing, and healthcare practices not routinely performed by Saudi healthcare students were among those excluded. No new material was added and the wording of retained items was not changed. The percentage of experts who rated each item as relevant (scored 3 or 4) was calculated as the Item-level Content Validity Index (I-CVI). Items with I-CVI below the acceptable threshold of 0.78 were examined and eliminated using expert consensus, items with I-CVI values >0.78 to 1.00 were retained. The content validity of the adapted questionnaire was then assessed using the Scale-level Content Validity Index/Average (S-CVI/Ave). Values of ≥ 0.90 were considered as excellent content validity.

After content validation, a pilot study was performed with 40 health professional students to evaluate the clarity, comprehensibility and time to complete the questionnaire. In order to test the reliability of the instrument over time, the same participants were asked to fill in the questionnaire again after a time lag of four weeks. Reliability analysis of the questionnaire was conducted using responses from the pilot study. The intra class correlation coefficient was observed to be 0.82 (good reliability). The awareness, attitude, and practice-based questions were considered to assess the questionnaire's internal consistency, as the knowledge component contained questions with multiple-response options, hence was not considered for the internal consitency. T Internal consistency was assessed separately for the awareness, attitude and practice domains, yielding cronbach's alpha values of 0.74, 0.72 and 0.77 respectively. Participants of the pilot study were excluded from the final analysis. Therefore, the adapted questionnaire demonstrated satisfactory content validity (S-CVI/Ave = 0.94).

### 2.6 Scoring of awareness, attitude and practice domains

The awareness domain comprised five scored items, with a possible total score of 0- 5. Scores of 4–5 (≥80%) were classified as high awareness, while scores of 0–3 were classified as low awareness. The attitude domain comprised six scored items, with a possible total score of 0- 6. Scores of 5–6 were classified as positive attitude, while scores of 0–4 were classified as negative attitude. The practice domain comprised five scored items, with a possible total score of 0- 5. Scores of 4–5 were classified as good practice, while scores of 0–3 were classified as poor practice. For each domain, responses meeting the predefined favorable/correct criterion were scored as 1, and other responses as 0, depending on the response format of the individual item.

### 2.7 Data collection procedure

The data were collected electronically using a secure online survey platform (Google Forms). The link to the survey was disseminated through university mailing lists, student organizations and official academic communication channels. Participation was anonymous and voluntary. Prior to commencing the survey, participants were given an electronic information sheet (Google Forms) that outlined the study's purpose, procedures, risks, benefits and confidentiality. The participants gave electronic consent before answering the questionnaire. To avoid duplicate responses, survey settings were configured to allow only one submission per participant. Data collection was open for approximately three months and every two weeks reminders were sent to encourage participation.

### 2.8 Statistical analysis and data management

Data collected through Google Forms was exported to Microsoft Excel (Microsoft Corp., New York, NY, USA), where data cleaning was performed. This process included checking for completeness, coding variables, and assessing missing data. In the present data there were no missing observations in the final analytical dataset, and all 456 participants were included in the analyses. This is further supported by the regression case-processing output, which indicated that all 456 cases were included in the analysis with zero missing cases. Consequently, no imputation, complete-case exclusion, or other missing-data procedure was required. Subsequently, the data were analyzed using the Statistical Package for the Social Sciences (SPSS) software, version 20 (2011; IBM Corp., Armonk, NY, USA).

Descriptive statistics were used to summarize the demographic characteristics of the participants in terms of frequency, percentage, and mean, where applicable. A two-step cluster analysis was conducted to categorize the participants based on their responses to knowledge-related questions. Two distinct knowledge clusters (high and low) were identified, after which inferential analysis was performed. The two-step cluster analysis was necessary because participants could select multiple responses for eight of the twelve knowledge questions. Conventional analysis of the total knowledge score would not have been appropriate in this context. Of the remaining four knowledge-based questions, three were single-response types and were independently dichotomized into correct and incorrect responses, while one question assessed confidence in lesion identification on a scale, which was dichotomized into high and low confidence.

Chi-square tests were applied to evaluate the association between independent demographic variables and the categorized knowledge, awareness, attitude, and practice domains. The interrelationships among these domains were also analyzed. Bivariate analyses were used to examine initial associations between the study variables and the categorized outcomes. Variables showing evidence of association and variables considered conceptually relevant to the respective outcomes were considered for multivariable binary logistic regression. Models were kept parsimonious in relation to the number of outcome events. Statistical significance was set at *p* < 0.05 for all analyses.

## 3 Results

A total of 456 participants were included in the final analysis. The mean age of the participants was 21.78 ± 3.73 years. The majority of responses were received from females, 352 (77.1%), compared to males, 104 (22.9%). Medical students constituted the largest professional group, with 280 (61.4%) participants, followed by applied sciences with 100 (21.9%), nursing with 53 (11.6%), and dentistry with 23 (5.0%). Most responses to the questionnaire were obtained from University A, 201 (44.1%), followed by University B, 90 (19.7%), University C, 74 (16.2%), University D, 63 (13.8%) and University E, 28 (6.1%). The details are presented in Table 1 and Supplementary Table S2.

**Table 1:** Demographic profile of the study participants.

Demographic Variable		Mean (± S.D)	Frequency (n)	Percentage (%)
Gender	Female	-	352	77.1
	Male	-	104	22.9
Age		21.78 (±3.73)	-	-
Professional Stream	Medical	-	280	61.4
	Applied Sciences	-	100	21.9
	Nursing		53	11.6
	Dental		23	5.0
Universities	University A	-	201	44.1
	University B	-	90	19.7
	University C	-	74	16.2
	University D		63	13.8
	University E	-	28	6.1

*N* = 456.

Two distinct knowledge clusters were identified using Two-Step cluster analysis. The Schwarz Bayesian Information Criterion (BIC) decreased substantially from 13647.513 for the one cluster solution to 12538.489 for two-cluster solution, representing a BIC change of −1109.023 and BIC ratio of 1.000. Although the absolute BIC continued to decrease with additional clusters, the magnitude of the relative improvement progressively diminished, with BIC change ratios of 0.380, 0.372,0.241, 0.203, and <0.10 for three, four, five, six and subsequent cluster solution respectively. The two- cluster solution also demonstrated the largest ratio of distance measure (2.185), indicating substantially greater separation between the two clusters [a lower knowledge cluster (*n* = 261) and a higher knowledge cluster (*n* = 195)] compared to other solutions. ‘The knowledge clusters were derived from 27 categorical response variables encompassing risk factors, oral changes, lesion recognition, and treatment-related responses.’. Participants in the higher knowledge cluster consistently demonstrated a greater ability to identify lesions, risk factors, oral changes, and appropriate treatment options. Specifically, leukoplakia was correctly identified by 160 (82.1%) of the higher knowledge cluster, tobacco use by 184 (94.4%), and non-healing ulcers by 131 (67.2%), compared to the lower knowledge cluster. The details are presented in Table 2.

**Table 2:** Characteristics of knowledge clusters identified by two-step cluster analysis.

Domain	Predictor	Lower Knowledge Cluster (*N* = 261) N and Percentage (%)	Higher Knowledge cluster (*N* = 195) N and Percentage (%)
Can you identify Lesions	Leukoplakia identifiable	34 (13.0)	160 (82.1)
	Erythroplakia identifiable	21 (8.0)	116 (59.5)
	Viral Infections identifiable	72 (27.6)	132 (67.7)
	Oral Lichen Planus identifiable	39 (14.9)	99 (50.8)
Can you identify Risk Factors	Tobacco use identifiable	152 (58.2)	184 (94.4)
	Smoking identifiable	153 (58.6)	183 (93.8)
	Smokeless tobacco identifiable	137 (52.5)	168 (86.2)
	Family history recognized	91 (34.9)	150 (76.9)
	Alcohol identifiable	117 (44.8)	135 (69.2)
Can you identify Oral changes	Non-healing ulcer identifiable	79 (30.3)	131 (67.2)
	White/red patches identifiable	87 (33.3)	101 (51.8)
Can you identify Treatment	Combination treatment identifiable	116 (44.4)	174 (89.2)

Knowledge level differed significantly according to the ability to correctly identify the age predilection for oral cancer (*p* < 0.001). Among participants who correctly identified the age predilection, 92 (63.4%) were classified in the higher knowledge cluster, compared to 53 (36.6%) in lower knowledge cluster. While those who incorrectly identified the age predilection for oral cancer, of them 103 (33.1%) were classified in the higher knowledge cluster and 208 (66.9%) respondents in lower knowledge cluster. The details are presented in Table 3.

**Table 3:** Chi-square analysis of the association between knowledge clusters and participants’ demographics.

Variable	Categories within variable	Higher Knowledge cluster n (%)	Lower Knowledge Cluster n (%)	Total n (%)	df	*p*-value
Age Group	17-22	123 (38.9)	193 (61.1)	316 (100)	1	0.013*
	≥23 years	72 (51.4)	68 (48.6)	140 (100)		
Professional Stream	Medical	143 (51.1)	137 (48.9)	280 (100)	3	<0.001*
	Applied Sciences	21 (21.0)	79 (79.0)	100 (100)		
	Nursing	13 (24.5)	40 (75.5)	53 (100)		
	Dental	18 (78.3)	5 (21.7)	23 (100)		
University	University A	90 (44.8)	111 (55.2)	201 (100)	4	0.057
	University B	43 (47.8)	47 (52.2)	90 (100)		
	University C	20 (27.0)	54 (73.0)	74 (100)		
	University D	29 (46.0)	34 (54.0)	63 (100)		
	University E	13 (46.4)	15 (53.6)	28 (100)		
Correct identification of common oral cancer site	Correct	59 (54.1)	50 (45.9)	109 (100)	1	0.006*
	Incorrect	136 (39.2)	211 (60.8)	347 (100)		
Correct identification of gender predilection	Correct	131 (59.3)	90 (40.7)	221 (100)	1	<0.001*
	Incorrect	64 (27.2)	171 (72.8)	235 (100)		
Correct identification of age predilection	Correct	92 (63.4)	53 (36.6)	145 (100)	1	<0.001*
	Incorrect	103 (33.1)	208 (66.9)	311 (100)		
Confidence in lesion identification	High Confidence	65 (46.4)	75 (53.6)	140 (100)	1	0.292
	Low Confidence	130 (41.1)	186 (58.9)	316 (100)		

Percentages in table are row percentages, * *P* < 0.05 - Significant.

The distribution of knowledge levels differed significantly by age group (*p* = 0.013), with 72 (51.4%) of 140 individuals in the older age group categorized in the higher knowledge cluster. A high proportion of dental students were observed in the higher knowledge cluster, and with significant variation in knowledge distribution among these students. The details are presented in Table 3.

There were no significant differences in knowledge between universities (*p* = 0.057). However, a significant association was observed between knowledge level and the correct identification of the common oral cancer site (*p* = 0.006), gender predilection (*p* < 0.001), and age predilection (*p* < 0.001). Knowledge level was not significantly associated with confidence in lesion identification (*p* = 0.292). The details are presented in Table 3.

Awareness levels differed significantly across the various professional streams (*p* = 0.006). Dental participants 15 (65.2%) exhibited the highest level of awareness. Participants in the higher knowledge category, 80 (41%) demonstrated high awareness while 115 (59%) demonstrated a significantly low level of awareness, compared to those in the lower knowledge category (*p* < 0.001). The details are presented in Table 4.

**Table 4:** Chi-square analysis of oPMDs and OC awareness, attitudes, and practices in relation to independent variables.

Awareness^a^						
Variable	Category	High Awareness	Low Awareness	Total	df	*p*-Value
Professional Stream	Medical	82 (29.3)	198 (70.7)	280 (100)	3	0.006*
	Applied Sciences	32 (32.0)	68 (68.0)	100 (100)		
	Nursing	17 (32.1)	36 (67.9)	53 (100)		
	Dental	15 (65.2)	8 (34.8)	23 (100)		
Knowledge Cluster	Higher Knowledge	80 (41.0)	115 (59.0)	195 (100)	1	<0.001*
	Low Knowledge	66 (25.3)	195 (74.7)	261 (100)		

Attitude^b^						
Variable	Category	Positive Attitude n (%)	Negative Attitude n (%)	Total n (%)	df	*p*-Value
Professional Stream	Medical	38 (13.6)	242 (86.4)	280 (100)	3	<0.001*
	Applied Sciences	17 (17.0)	83 (83.0)	100 (100)		
	Nursing	13 (24.5)	40 (75.5)	53 (100)		
	Dental	12 (52.2)	11 (47.8)	23 (100)		
University	University A	27 (13.4)	174 (86.6)	201 (100)	4	0.002*
	University B	17 (18.9)	73 (81.1)	90 (100)		
	University C	16 (21.6)	58 (78.4)	74 (100)		
	University D	8 (12.7)	55 (87.3)	63 (100)		
	University E	12 (42.9)	16 (57.1)	28 (100)		
Knowledge Cluster	Higher Knowledge cluster	42 (21.5)	153 (78.5)	195 (100)	1	0.053
	Lower Knowledge cluster	38 (14.6)	223 (85.4)	261 (100)		

Percentages in table are row percentages, *P < 0.05 – Significant, ^a^High awareness (4-5) and Low Awareness (0-3), ^b^Positive attitude (5-6) and Negative Attitude (0-4).

Attitude levels, when analyzed, showed significant variation across professional streams (*p* < 0.001). Similar to awareness, participants from the dental profession—12 individuals (52.2%)—had the highest proportion of positive attitudes. Approximately 12 (42.9%) participants from University E, exhibited a higher proportion of positive attitudes compared to participants from universities with major representation. Although a greater proportion of participants with higher knowledge levels 42 (21.5%) exhibited a positive attitude compared to those with lower knowledge levels, this association was not statistically significant (*p* = 0.053). The details are presented in Table 4.

Similarly, regarding awareness and attitude, good practice was significantly associated with professional stream (*p* < 0.001), awareness level (*p* < 0.001), and attitude level (*p* < 0.001). Among dental participants, 15 (65.2%) demonstrated the highest proportion of good practice. Of those categorized as having high awareness, 56 (38.4%) exhibited a greater proportion of good practices. Similarly, participants with positive attitudes demonstrated a greater proportion of good practice compared to those with negative attitudes. No significant association was observed between good practice and knowledge levels (*p* = 0.374). The details are presented in Table 5.

**Table 5:** Chi-square analysis of practices related to the clinical assessment of oPMDs and OC with independent variables. .

Practice						
Variable	Category	Good Practice**^a^**	Poor Practice**^a^**	Total	df	*p*-Value
Professional Stream	Medical	46 (16.4)	234 (83.6)	280 (100)	3	<0.001*
	Applied Sciences	19 (19.0)	81 (81.0)	100 (100)		
	Nursing	14 (26.4)	39 (73.6)	53 (100)		
	Dental	15 (65.2)	8 (34.8)	23 (100)		
Awareness Level	High Awareness	56 (38.4)	90 (61.6)	146 (100)	1	<0.001*
	Low Awareness	38 (12.3)	272 (87.7)	310 (100)		
Attitude Level	Positive Attitude	42 (52.5)	38 (47.5)	80 (100)	1	<0.001*
	Negative Attitude	52 (13.8)	324 (86.2)	376 (100)		
Knowledge Cluster	Higher Knowledge	44 (22.6)	151 (77.4)	195 (100)	1	0.374
	Low Knowledge	50 (19.2)	211 (80.8)	261 (100)		

Percentages in table are row percentages, ^a^Good practice (total score of 4-5) compared to poor practice (total score of 0 −3), *P < 0.05 - Significant.

In the regression analysis, the positive-attitude model was significant overall [*χ*^2^(8) = 32.48, *p* < 0.001], with a −2 Log Likelihood of 391.06, Cox–Snell R^2^ of 0.069 and Nagelkerke R^2^ of 0.114; the Hosmer–Lemeshow test was non-significant [*χ*^2^(7) = 3.73, *p* = 0.811]. The higher-knowledge model was also significant overall [*χ*^2^(4) = 49.45, *p* < 0.001], with a −2 Log Likelihood of 573.11, Cox–Snell R^2^ of 0.103 and Nagelkerke R^2^ of 0.138; the Hosmer–Lemeshow test was non-significant [*χ*^2^(4) = 0.214, *p* = 0.995]. The high-awareness logistic regression model was statistically significant overall [likelihood-ratio *χ*^2^(4) = 22.91, *p* < 0.001], with a −2 Log Likelihood of 548.92, Cox–Snell R^2^ of 0.049 and Nagelkerke R^2^ of 0.069. The Hosmer–Lemeshow test was non-significant [*χ*^2^(3) = 0.548, *p* = 0.908]. The good-practice logistic regression model was statistically significant overall [*χ*^2^(5) = 88.08, *p* < 0.001], with a −2 Log Likelihood of 375.94, Cox–Snell R^2^ of 0.176, and Nagelkerke R^2^ of 0.275. The Hosmer–Lemeshow test was non-significant [*χ*^2^(5) = 6.814, *p* = 0.235], indicating no evidence of lack of fit.

Participants from the dental (OR = 12.67, 95% CI: 4.17–38.4, *p* < 0.001) and medical (OR = 3.77, 95% CI: 2.19–6.49, *p* < 0.001) professional streams demonstrated significantly higher odds of belonging to the higher knowledge category compared with participants from applied sciences. There was no significant difference in knowledge between the nursing and applied sciences professions (OR = 1.23, 95% CI:0.55-2.71,*p* = 0.611). Age group was also observed to be a non-significant predictor of knowledge levels (OR =1.22, 95% CI: 0.79-1.87, *p* = 0.366). As a sensitivity analysis, age was additionally modeled as a continuous variable in the higher-knowledge model, and the association remained non-significant (OR = 0.999, 95% CI: 0.944–1.057, *p* = 0.973). The details are presented in Table 6.

**Table 6:** Binary logistic regression analysis of predictors associated with higher knowledge and awareness.

Outcome	Independent Variable	Category	B	OR	Confidence Interval (95%)		*p*-Value
					**Lower**	**Upper**	
Higher Knowledge Cluster	Professional Stream	Dental	2.54	12.67	4.17	38.4	<0.001*
		Medical	1.32	3.77	2.19	6.49	<0.001*
		Nursing	0.20	1.23	0.55	2.71	0.611
		Ref: Applied Sciences	-	-	-	-	-
	Age Group	≥23 years	0.20	1.22	0.79	1.87	0.366
		Ref: 17-22 years	-	-	-	-	-
HighAwareness	Professional Stream	Dental	0.98	2.68	0.99	7.23	0.052
		Medical	-0.36	0.69	0.41	1.17	0.165
		Nursing	-0.02	0.97	0.47	2.01	0.935
		Ref: Applied Sciences	-	-	-	-	-
	Knowledge Cluster	Higher	0.73	2.07	1.35	3.19	0.001*
		Ref: Lower	-	-	-	-	-

*P < 0.05 – Significant.

Higher knowledge was a significant predictor of awareness level. Participants with higher knowledge (OR = 2.07, 95% CI: 1.35–3.19, *p* = 0.001) had significantly greater odds of having high awareness compared to those in the lower knowledge category. Although dental participants exhibited higher awareness compared to other professional streams, this difference was not statistically significant (OR =  2.68, 95% CI: 0.99-7.23, *p* = 0.052), as observed in the regression analysis. The details are presented in Table 6.

Although the association between the knowledge cluster and positive attitude was borderline in the bivariate analysis (*p* = 0.053), we retained the knowledge cluster in the multivariable model because it is conceptually relevant to attitude formation and showed borderline significance. Professional stream and university affiliation were significant predictors of a positive attitude. Among the professional students, participants from the dental students (OR = 3.54, 95% CI: 1.23–10.17, *p* = 0.019) demonstrated significantly higher odds of a positive attitude toward oral cancer examination, screening, and education compared to participants from the applied sciences profession. Medical students (OR = 0.62, 95% CI: 0.30–1.24, *p* = 0.187) had lower odds of a positive attitude compared to those from the applied sciences. Although participants in the higher knowledge category showed greater odds of a positive attitude than those in the lower knowledge category, this difference was not statistically significant. The details are presented in Table 7.

**Table 7:** Binary logistic regression analysis of predictors associated with attitude and practice.

Outcome	Independent Variable	Category	B	OR	Confidence Interval (95%)		*p*-Value
					**Lower**	**Upper**	
Positive Attitude	Professional Stream	Dental	1.26	3.54	1.23	10.17	0.019*
		Medical	−0.47	0.62	0.30	1.24	0.187
		Nursing	0.13	1.14	0.46	2.84	0.778
		Ref: Applied Sciences	-	-	-	-	-
	Knowledge Cluster	Higher	0.50	1.66	0.96	2.87	0.070
		Ref: Lower	-	-	-	-	-
	University	University A	0.06	1.06	0.45	2.48	0.894
		University B	0.47	1.60	0.64	3.97	0.313
		University C	0.64	1.89	0.75	4.78	0.178
		University D	1.64	5.15	1.79	14.78	0.002*
		Ref: University E	-	-	-	-	-
Good Practice	Professional Stream	Dental	1.48	4.40	1.44	13.43	0.009*
		Medical	−0.10	0.90	0.47	1.71	0.749
		Nursing	0.38	1.46	0.62	3.44	0.387
		Ref: Applied Sciences	-	-	-	-	-
	Awareness	High Awareness	1.26	3.53	2.11	5.92	<0.001*
		Ref: Low Awareness	-	-	-	-	-
	Attitude	Positive	1.62	5.10	2.89	8.98	<0.001*
		Ref: Negative	-	-	-	-	-

*P < 0.05 – Significant.

Participants with high awareness (OR = 3.53, 95% CI: 2.11–5.92, *p* < 0.001) and positive attitudes (OR = 5.10, 95% CI: 2.90–8.98, *p* < 0.001) had significantly higher odds of good practice. Professional stream was also associated with good practice. Compared with students from Applied Sciences, dental students had higher odds of good practice (OR = 4.40, 95% CI: 1.44–13.43, *p* = 0.009), whereas the associations for medical students (OR = 0.90, 95% CI: 0.47–1.71, *p* = 0.750) and nursing students (OR = 1.46, 95% CI: 0.62–3.44, *p* = 0.387) were not statistically significant. The details are presented in Table 7.

Model analysis included assessing multicollinearity and influential observations using variance inflation factors, Cook's distance, leverage, and DFBETAs; the diagnostic findings were within acceptable limits.

## 4 Discussion

The primary purpose of this study was to assess the knowledge, awareness, attitudes and practices of health professional students towards OPMDs and OC in Riyadh, Saudi Arabia. The results showed that there were obvious differences between the dental students and other disciplines, where dental students had the highest level of knowledge, awareness, attitudes and practices (KAAP) compared to medical, nursing and applied medical sciences students. Findings indicate gaps in their KAAP that may impact the future healthcare workforce's ability to meaningfully contribute to OC prevention and early screening.

Medical and other healthcare professionals play a crucial role in identifying potential oral cancers, especially in high-risk patients. Incorporating oral examinations into regular physical check-ups helps integrate medical and dental care, fostering a more unified approach to patient treatment (17, 18). Collaboration across disciplines, involving dental and other healthcare experts, can improve oral cancer screening. Ongoing education, cross-disciplinary training, and the use of shared electronic health records enhance communication and facilitate earlier detection (19).

In the present study, two distinct knowledge clusters were identified. Participants in the higher knowledge cluster demonstrated significantly greater awareness of OPMDs, risk factors for oral cancer, clinical manifestations, and treatment approaches. The results of the present study were in line with findings reported in previous literature (12, 20). While recognition of tobacco-related risk factors was relatively high, awareness of specific OPMDs and early clinical signs was suboptimal among many participants. Similar results have been reported worldwide, showing that healthcare students generally acknowledge tobacco as a major risk factor but possess limited knowledge of potentially malignant disorders and early signs of oral cancer. This is consistent with the findings of Sharma, P. et al. (21), who found that dental students had sufficient knowledge regarding the prevention and detection of oral cancer, whereas medical students were less aware of investigative methods for oral cancer. Our results contrast with the study by Petrauskienė S. et al. (22), which reported that international dental and medical students had fair self-reported knowledge of OC risk factors, with medical students demonstrating better knowledge about OC risk factors than dental students. These discrepancies may reflect variations in educational curricula, academic stages, clinical exposure, prior training, study populations, and questionnaire content across different settings. However, these factors were not assessed in the present study; therefore, the reasons for the observed differences cannot be determined.

The dental subgroup showed higher proportions of knowledge, awareness, positive attitudes, and good clinical practices compared with students from other professional disciplines. However, these findings should be interpreted cautiously because only 23 dental students (5.0%) were included. The observed odds ratio for higher knowledge (OR = 12.67, 95% CI: 4.17–38.40) has a wide confidence interval, indicating considerable uncertainty in the estimate. Thus, these findings should be regarded as exploratory rather than definitive and require confirmation in larger studies with balanced representation across professional disciplines. The observed differences may be related to greater exposure to oral pathology, oral medicine, oral diagnosis, and oral cancer screening within dental curricula and clinical training; however, these explanations were not directly assessed in the present study (23, 24). Nevertheless, the small and uneven subgroup sizes limit the robustness and generalizability of these comparisons; therefore, the findings should be considered exploratory.

Furthermore, participants in the higher knowledge cluster had significantly greater odds of demonstrating high awareness, highlighting the strong relationship between knowledge acquisition and awareness. Additionally, participants with positive attitudes were significantly more likely to demonstrate good clinical practice, emphasizing the importance of fostering positive attitudes alongside knowledge to enhance clinical preparedness.

The sample distribution across professional disciplines was markedly uneven, with dental students comprising only 5.0% of the sample, compared to 61.4% from medicine, 21.9% from applied sciences, and 11.6% from nursing. Consequently, estimates involving dental students—particularly the observed odds ratios—should be interpreted with caution due to the relatively small subgroup size and the associated uncertainty, as indicated by the wide confidence intervals. Additionally, participant numbers varied significantly across universities. Since professional disciplines may be unevenly distributed across institutions and geographic regions, the effects attributed to professional discipline may partially reflect institutional or contextual differences. Therefore, the observed association between professional discipline and knowledge should not be interpreted as a purely discipline-specific effect.

Previous studies conducted both internationally and in Saudi Arabia have consistently reported better OC knowledge among dental students compared to students of medicine, nursing, pharmacy, and other health-related disciplines. Kujan et al. reported generally satisfactory awareness among Saudi dental students regarding OC risk factors and clinical manifestations, although some knowledge gaps remained (11). Escoto Vasquez et al. (14) recently concluded in a systematic review that despite increased educational exposure to topics related to cancer prevention, significant gaps in knowledge of OC persist among health professional students.

Another important finding of this study was the significant relationship between knowledge and awareness. Students in the high-knowledge cluster were nearly twice as likely to be highly aware compared to those in the low-knowledge cluster. This finding supports the theoretical framework in health professional education, which posits that knowledge is a prerequisite for awareness and informed clinical decision-making. However, improved clinical practice was not independently associated with increased knowledge. This suggests that knowledge alone is not always sufficient to translate into effective clinical behavior, highlighting the importance of experiential learning, clinical exposure, and opportunities for practical training. Similar findings have been reported among healthcare students and professionals, where theoretical knowledge did not consistently translate into confidence in performing oral examinations or referring patients with suspicious lesions (25, 26).

In the present study, awareness and a positive attitude were also found to be significant predictors of good practice. Participants with high awareness were more than three times as likely to report good clinical practices, while those with positive attitudes were more than five times as likely to exhibit such practices. These findings align with behavioral theories suggesting that knowledge influences awareness, which in turn shapes attitudes and ultimately behavior (27, 28). Similar associations between awareness, attitudes, and screening practices have been reported among healthcare students and practitioners involved in OC prevention and early screening programs (13, 25).

In the present study, medical students demonstrated better knowledge than students of applied medical sciences; however, their attitudes and practices did not appear to be significantly better. This finding may reflect limited exposure to oral health education in the undergraduate medical curriculum. Previous studies have shown that physicians often encounter patients with oral lesions during routine clinical care but report insufficient training in oral examination and OC screening (29, 30). Medical practitioners are often the first contact in healthcare systems, and thus, enhancement of OC education in medical curricula can contribute significantly to earlier detection and referral of suspicious lesions.

The observed differences across professional disciplines may reflect differences in educational curricula, academic progression, prior training, or clinical exposure; however, these factors were not assessed in the present study. Therefore, it is not possible to determine whether the observed differences were attributable to professional discipline itself or to differences in educational and clinical exposure. The findings should consequently be interpreted as associations within the study sample rather than evidence of an independent effect of professional discipline.

Another finding was the absence of a statistically significant difference in knowledge among the participating universities. The university groups varied substantially in size, ranging from 28 to 201 participants. This imbalance may have led to unequal precision in institutional estimates and could have limited the ability to detect differences between universities. Additionally, the association between university and knowledge was not statistically significant at the conventional threshold (*p* = 0.057); however, this borderline result should not be interpreted as evidence of equivalent knowledge levels across institutions. Potential within-institution correlation was also not formally modeled. Therefore, university-level comparisons should be considered exploratory and interpreted with caution. Future studies should employ more balanced institutional sampling and statistical methods that account for clustering within institutions. Furthermore, interprofessional education strategies, such as collaborative learning among dental, medical, nursing, and allied health students, may be particularly useful in enhancing knowledge of OC prevention and early detection (31).

Healthcare professionals play an important role in personalized screening, patient counseling, tobacco cessation interventions, and standardized referral processes. Therefore, competent future healthcare professionals are needed to improve patient outcomes and strengthen national OC control strategies. Early identification of OPMDs is still one of the most effective methods to reduce OC morbidity and mortality, thus highlighting the importance of healthcare workforce readiness (32, 33).

### 4.1 Clinical relevance

The findings highlight the importance of enhancing education on oral cancer and OPMD education within healthcare professional curricula. Targeted educational initiatives focusing on risk factors, early detection of suspicious oral lesions, prevention, and appropriate referral can help address existing knowledge and practice gaps. Educators and curriculum planners should consider integrating interdisciplinary, clinically oriented oral cancer education across healthcare programs. Additionally, policymakers and institutions could support standardized educational programs and continuing professional development to promote early recognition and timely referral of potentially malignant and malignant oral lesions. Given the exploratory nature of some subgroup findings, particularly among dental students, these implications warrant further evaluation in larger, more representative studies.

### 4.2 Strengths and limitations

Strengths of this study include its multicenter design, the inclusion of diverse healthcare disciplines, and the assessment of multiple domains, including knowledge, awareness, attitudes, and practices. However, there are some limitations: the cross-sectional nature of the study does not allow for causal inference, and the self-reported data are susceptible to recall and social desirability biases and the unequal distribution of participants across professional streams, which may limit generalizability. Selection and nonresponse bias are important considerations in this study. The survey employed voluntary online convenience sampling, which may have led to the self-selection of students who were more interested in oral health, OPMDs, or oral cancer. This selection bias could have contributed to higher observed knowledge levels or more favorable awareness, attitude, and practice scores than those present in the broader student population. Additionally, because a complete sampling frame and denominator of eligible students were not available, a reliable response rate could not be calculated, and the extent of nonresponse bias could not be formally assessed. Therefore, the representativeness of the sample cannot be established, and the findings should be interpreted cautiously, primarily as associations observed among the participating students. Another limitation is that the academic year and clinical/preclinical status, prior formal training in oral cancer or OPMDs, and the extent of previous clinical exposure to patients with oral mucosal lesions were not assessed in this study. These factors may influence knowledge, awareness, attitudes, and practices and could partly explain the observed differences between professional groups. However, because these variables were not collected, their potential confounding effects could not be evaluated or adjusted for statistically. Therefore, the observed differences between professional disciplines should not be attributed solely to discipline. Future research should include these variables to enable more meaningful comparisons across professional disciplines and educational levels. The sample was also substantially imbalanced across professional disciplines and universities, with only 23 dental students (5.0%), limiting the precision and generalizability of comparisons involving this subgroup. The marked imbalance in sex distribution may represent an additional source of selection bias. Finally, the study was conducted exclusively among healthcare students in Riyadh, and the findings should not be generalized to healthcare students throughout Saudi Arabia. The results should be interpreted specifically within the context of the participating students and institutions in Riyadh. Larger, multicenter studies employing probability-based sampling, balanced representation across professional disciplines and institutions, validated measurement instruments, objective assessments of clinical performance, and adjustments for relevant educational and demographic confounders are warranted to confirm and extend these findings. An additional limitation is that potential correlation within institutions was not formally accounted for in the analyses. Although university was included as an explanatory variable in the relevant analyses, this does not fully account for the hierarchical structure of the data. Consequently, standard errors and effect estimates may not adequately reflect potential within-institution dependence, and associations involving professional discipline and university should be interpreted as exploratory. Future studies should employ sampling designs that ensure adequate representation within institutions and apply appropriate statistical methods, such as multilevel logistic regression, generalized estimating equations, or cluster-robust techniques to account for institutional clustering. Despite these limitations, the study provides valuable baseline information on the educational readiness of Saudi Arabian healthcare students on OC and OPMDs.

## 5 Conclusions

Among health professional students in Riyadh, the findings demonstrated variation in knowledge, awareness, attitudes, and practices across professional groups, with dental students showing higher proportions of favorable responses in several domains. The dental subgroup showed higher knowledge and more positive responses than students from other professional disciplines; however, these findings are exploratory and imprecise given the small dental subgroup (*n* = 23) and wide confidence intervals. They should therefore not be interpreted as definitive evidence of discipline-specific superiority and require confirmation in larger, balanced studies. No significant differences in knowledge were observed across the participating universities; nevertheless, this does not confirm that educational gaps are systemic rather than institution specific. These findings underscore the importance of enhancing oral cancer education, practical training opportunities, and interprofessional collaboration within healthcare curricula. Further multicenter studies employing representative sampling, validated assessment tools, and objective measures of clinical performance are necessary to assess healthcare students' preparedness and to evaluate the effectiveness of educational interventions in improving oral cancer prevention and early detection. Improving the awareness of future health care professional students may potentially help them to recognize and refer suspicious oral lesions earlier, leading to improved patient outcomes and reducing burden of oral cancer (34).

## Data Availability

The original contributions presented in the study are included in the article/Supplementary Material, further inquiries can be directed to the corresponding author.
