# Supplementary material for: Knowledge, awareness, attitudes and practice regarding oral potentially malignant disorders and oral cancer among health professional students in Riyadh, Saudi Arabia: a multicenter cross-sectional study
Source: Front Oral Health. 2026 Oct 21;7:1942387. doi: 10.3389/froh.2026.1942387 (PMC13731213; doi:10.3389/froh.2026.1942387)
Supplement: Supplementary file 1 [file Table1.docx]

**Supplementary Table S1**: STROBE Checklist for Cross-Sectional Studies

| **STROBE item** | **Recommendation** | **Location in revised manuscript** |
| --- | --- | --- |
| **Title and abstract** |  |  |
| **1a. Title** | Indicate the study design with a commonly used term | Title; identifies the study as a “Multicenter Cross-Sectional Study” |
| **1b. Abstract** | Provide an informative and balanced summary of what was done and what was found | Abstract |
| **Introduction** |  |  |
| **2. Background/rationale** | Explain the scientific background and rationale for the investigation | Introduction, paragraphs 1–4 |
| **3. Objectives** | State specific objectives, including any prespecified hypotheses | Final paragraph of Introduction |
| **Methods** |  |  |
| **4. Study design** | Present key elements of study design early in the manuscript | Section 2.1: Research Design and Setting |
| **5. Setting** | Describe setting, locations, and relevant dates, including recruitment and data collection periods | Section 2.1: Research Design and Setting |
| **6a. Participants** | Give eligibility criteria and sources and methods of selection of participants | Sections 2.3 and 2.4: Sampling Technique and Eligibility Criteria  [ Students were recruited by **voluntary online convenience sampling]** |
| **6b. Matching** | If applicable, give matching criteria and numbers of exposed/unexposed | Not applicable; no matching was performed |
| **7. Variables** | Clearly define all outcomes, exposures, predictors, potential confounders, and effect modifiers | Sections 2.5 and 2.7; Tables 1–7 |
| **8. Data sources/measurement** | For each variable, give sources and methods of assessment; describe comparability of assessment methods | Section 2.5: Data Collection Tool; Section 2.6: Data Collection Procedure |
| **9. Bias** | Describe efforts to address potential sources of bias | Sections 2.3, 2.5, 2.6, and Limitations |
| **10. Study size** | Explain how study size was arrived at | Section 2.2: Sample Size Estimation |
| **11. Quantitative variables** | Explain how quantitative variables were handled in analyses and, if applicable, which groupings were chosen and why | Sections 2.7 and Results; age categorized as 17–22 and ≥23 years; categorized knowledge, awareness, attitude, and practice outcomes |
| **12a. Statistical methods** | Describe all statistical methods, including those used to control for confounding | Section 2.7: Statistical Analysis and Data Management |
| **12b. Subgroups/interactions** | Describe any methods used to examine subgroups and interactions | Section 2.7; analyses stratified by professional stream, university, age group, and knowledge/awareness/attitude categories |
| **12c. Missing data** | Explain how missing data were addressed | Section 2.7: data cleaning included assessment of completeness and missing data. However, there There were no missing observations in the final analytical dataset, and all 456 participants were included in the analyses |
| **12d. Loss to follow-up** | If applicable, describe methods addressing loss to follow-up | Not applicable; cross-sectional study with no follow-up |
| **12e. Sensitivity analyses** | Describe any sensitivity analyses | No sensitivity analyses were performed |
| **Results** |  |  |
| **13a. Participants—numbers** | Report numbers of individuals at each stage of study | Results: 456 participants included in final analysis |
| **13b. Reasons for non-participation** | Give reasons for non-participation at each stage | Not available because participation was voluntary and the response denominator was unavailable  [Not available; an overall response rate could not be calculated because the number of students who received the survey link was not recorded] |
| **13c. Flow diagram** | Consider use of a flow diagram | Not provided; recruitment was voluntary online and the number invited could not be determined |
| **14a. Descriptive data** | Give characteristics of study participants and information on exposures/confounders | Table 1; Results |
| **14b. Missing data** | Indicate number of participants with missing data for each variable of interest | Results/Statistical Analysis; missing data were assessed during data cleaning [There were no missing observations in the final analytical dataset, and all 456 participants were included in the analyses. This is further supported by the regression case-processing output, which indicated that all 456 cases were included in the analysis with zero missing cases. Consequently, no imputation, complete-case exclusion, or other missing-data procedure was required.] |
| **14c. Outcome data** | Report numbers of outcome events or summary measures | Tables 2–7; Results |
| **15. Main results** | Give unadjusted estimates and, where applicable, confounder-adjusted estimates with precision | Tables 3–7; odds ratios with 95% CIs reported in Tables 6 and 7 |
| **16a. Other analyses** | Report other analyses, including subgroup analyses and sensitivity analyses | Tables 2–5; two-step cluster analysis and chi-square analyses |
| **16b. Category boundaries** | Report category boundaries when continuous variables were categorized | Results and Tables 3–5; age groups and categorized knowledge, awareness, attitude, and practice |
| **Discussion** |  |  |
| **17. Key results** | Summarize key results with reference to study objectives | First paragraphs of Discussion |
| **18. Limitations** | Discuss limitations, including potential sources of bias or imprecision | Limitations paragraph(s) in Discussion |
| **19. Interpretation** | Give a cautious overall interpretation considering objectives, limitations, multiplicity of analyses, and other evidence | Discussion |
| **20. Generalizability** | Discuss external validity/generalizability | Discussion/Limitations; findings restricted to participating healthcare students in Riyadh |
| **Other information** |  |  |
| **21. Funding** | Give source of funding and role of funders | Funding statement |
| **22. Ethics** | Give ethical approval and informed consent where applicable | Section 2.1; Ethics Statement |

**Supplementary Table S2:** Cross tabulation to assess responses from professional stream from various universities

| Variable | Category | **University** | | | | | Total | df | Fishers  p-Value |
| --- | --- | --- | --- | --- | --- | --- | --- | --- | --- |
|  |  | A | B | C | D | E |  |  |  |
| Professional Stream | Applied Sciences | 51  (51.0) | 39  (39.0) | 3  (3.0) | 1  (1.0) | 6  (6.0) | 100 (100) | 12 | <0.001* |
|  | Dental | 9  (39.1) | 6  (26.1) | 5  (21.7) | 0  (0.0) | 3  (13.0) | 23 (100) |  |  |
|  | Medical | 121 (43.2) | 45  (16.1) | 40  (14.3) | 62  (22.1) | 12  (4.3) | 280 (100) |  |  |
|  | Nursing | 20  (37.7) | 0  (0.0) | 26  (49.1) | 0  (0.0) | 7  (13.2) | 53 (100) |  |  |
| Total | | 201 (44.1) | 90  (19.7) | 74  (16.2) | 63  (13.8) | 28  (6.1) | 456 (100) |  |  |
